# Correction: Human IgA bind a diverse array of commensal bacteria

**DOI:** 10.1084/jem.2018163501152020c

**Published:** 2020-01-28

**Authors:** Delphine Sterlin, Jehane Fadlallah, Olivia Adams, Claire Fieschi, Christophe Parizot, Karim Dorgham, Asok Rajkumar, Gaëlle Autaa, Hela El-Kafsi, Jean-Luc Charuel, Catherine Juste, Friederike Jönsson, Thomas Candela, Hedda Wardemann, Alexandra Aubry, Carmen Capito, Hélène Brisson, Christophe Tresallet, Richard D. Cummings, Martin Larsen, Hans Yssel, Stephan von Gunten, Guy Gorochov

Vol. 217, No. 3, March 2, 2020. 10.1084/jem.20181635

*JEM* regrets that in the original version of this paper, the x- and y-axes labels in Fig. 1 E were missing as a result of a conversion error. In addition, “colon” and “ileum” labels have been added to Fig. S1 D, and its legend has been edited for clarity. The corrected figures and legends appear below. All versions of this article have been corrected.

**Figure 1.**
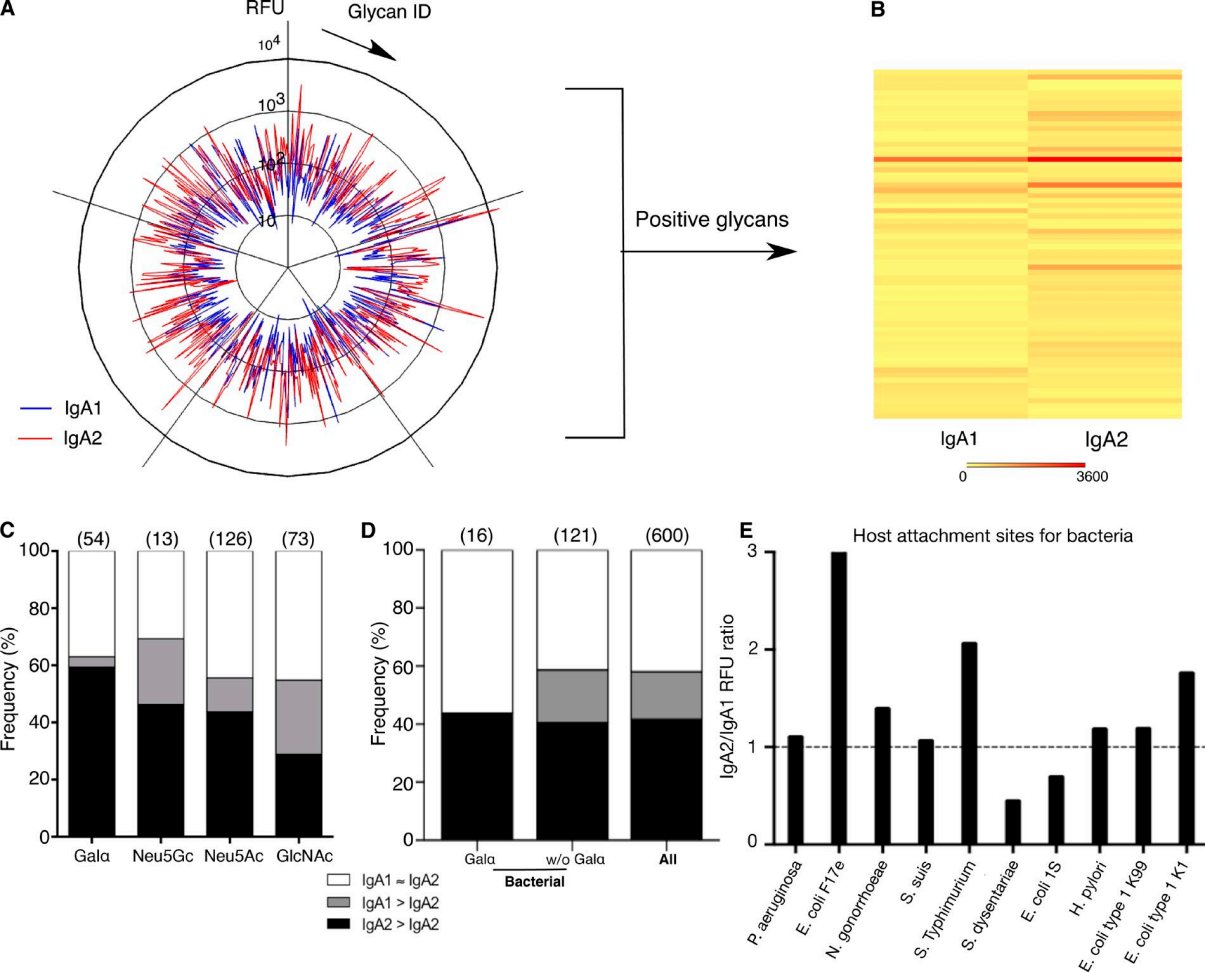
**Carbohydrate-binding profile of polyclonal IgA1 and IgA2 antibodies. (A)** Glycan reactivity of serum polyclonal IgA1 and IgA2 (*n* = 5 healthy donors pooled in one experiment). Each peak represents an individual glycan recognized by IgA1 (blue line) or IgA2 (red line). **(B)** Heatmap diagram depicting glycans recognized by IgA1 or IgA2. Each row represents an individual glycan. **(C)** Preferential recognition of distinct terminal carbohydrate moieties by serum polyclonal IgA1, IgA2, or both. Terminal carbohydrate moieties equally recognized by both IgA1 and IgA2 are depicted in white. Terminal moieties preferentially recognized by IgA1 or IgA2 are showed in gray or black, respectively. **(D)** Isotype-dependent recognition of Galα-terminal structures of bacterial origin and other bacterial antigens excluding Galα-terminal antigens (without Galα) by serum polyclonal IgA, as compared with entire glycan microarray dataset. Terminal carbohydrate moieties equally recognized by both IgA1 and IgA2 are depicted in white. Terminal moieties preferentially recognized by IgA1 or IgA2 are showed in gray or black, respectively. **(E)** Differential isotype binding to bacterial attachment sites.

**Figure S1.**
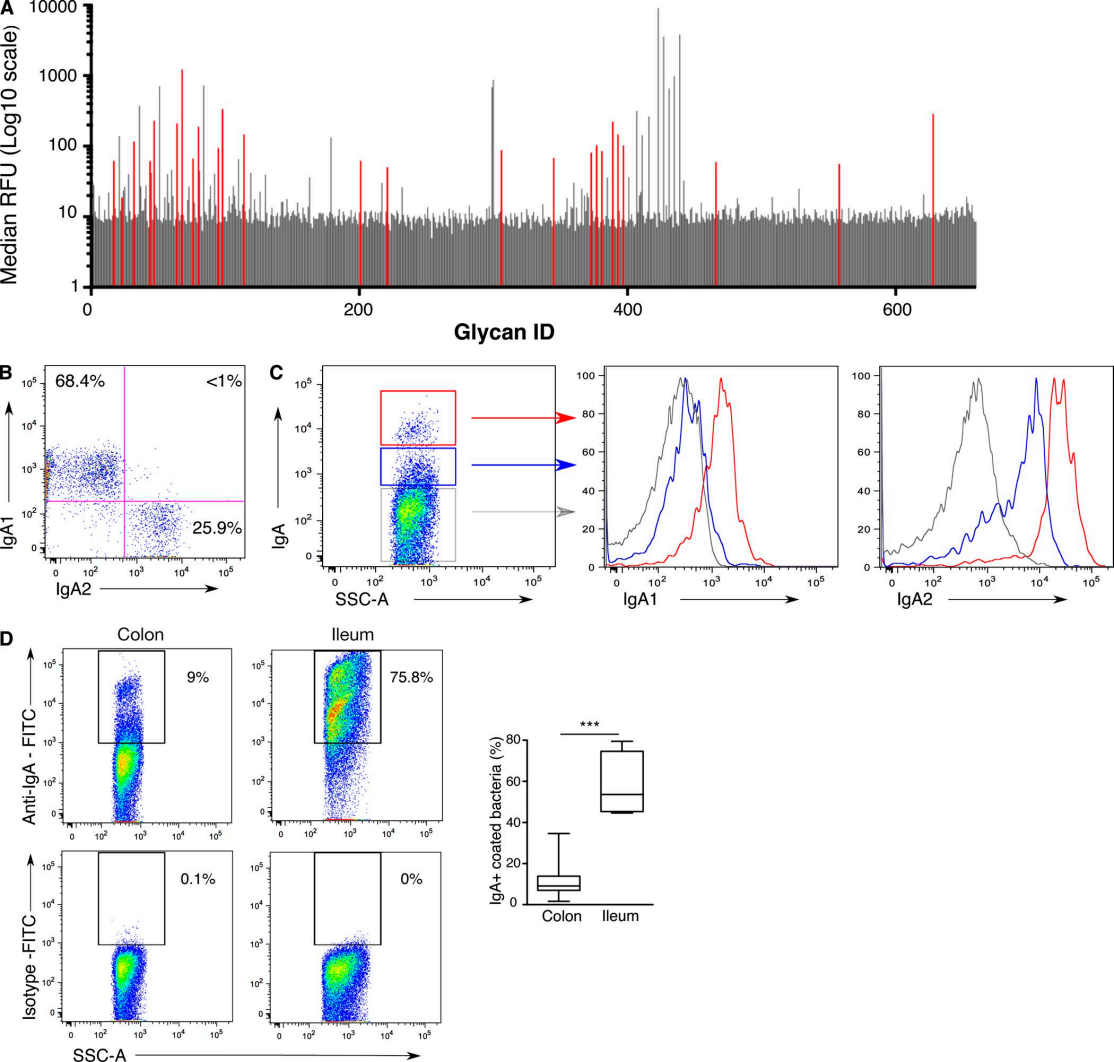
**Gut bacteria segregate into IgA^bright^ and IgA^low^ fractions in healthy humans.** Related to Figs. 1 and 2. **(A)** The secretory component binds a modest range of carbohydrates. Glycan reactivity with the secretory component was assessed using glycan microarray technology (660 structures). Representative median RFUs are shown. Glycans specifically recognized by secretory component are colored in red. **(B)** Anti-IgA1 and anti-IgA2 antibodies do not cross-react. Flow cytometry analysis of IgA1 and IgA2 expression on peripheral B cells from a healthy donor. **(C)** IgA-coated bacteria split into IgA^bright^ and IgA^low^ fractions depending on IgA1 and IgA2 coating. Representative flow cytometry analysis of IgA1 and IgA2 coating in IgA^bright^-coated bacteria (red lines), IgA^low^-coated bacteria (blue lines), and IgA-unbound bacteria (gray lines). **(D)** Representative flow cytometric analysis of colon and ileum microbiota (left and central panels, respectively) with anti-IgA FITC or isotype-matched control antibody, as indicated. Numbers indicate percentage of positive cells. Data are cumulative from three independent experiments. Boxes extend from the 25th to the 75th percentiles. Error bars represent minimum and maximum values. P values were defined using the Mann-Whitney test. ***, P < 0.001. SSC-A, side scatter area. Quantification of IgA-coated bacteria in stool (*n* = 20) and ileum (*n* = 5).

